# Simultaneous realization of polarization conversion for reflected and transmitted waves with bi-functional metasurface

**DOI:** 10.1038/s41598-022-06366-6

**Published:** 2022-02-11

**Authors:** Xiaojun Huang, Xia Ma, Xuewen Li, Jingdao Fan, Liang Guo, Helin Yang

**Affiliations:** 1 College of Communication and Information Engineering, Xi’an University of Science and Technology, Xi’an, 710054 China; 2 College of Safety Science and Engineering, Xi’an University of Science and Technology, Xi’an, 710054 Shaanxi China; 3 College of Physics and Electrical Engineering, Kashi University, Kashi, 844007 China; 4 College of Physical Science and Technology, Central China Normal University, Wuhan, 430079 China

**Keywords:** Electronic devices, Metamaterials, Microwave photonics

## Abstract

Manipulating the polarizations of electroagnetic waves by flexible and diverse means is desirable for myriad microwave systems. More recently, metasurfaces have emerged as promising alternatives to conventional polarization manipulation components because the flexibility of their geometry means that they can be arbitrarily customized. In this context, a bilayered metasurface is presented to simultaneously manipulate the polarized states of reflected and transmitted microwaves. Regardless of whether an incident electromagnetic wave is x-polarized or y-polarized, the reflected and transmitted waves are converted into their orthogonal waves at the operating frequency. The designed metasurface has a high polarization conversion rate, above 90%, for both normal and oblique incidences. Experimental results verify the correctness of the simulated results. Finally, the axial ratio and surface current distributions are employed to reveal the physics of the polarization manipulation. The proposed metasurface will be beneficial in the design of flexible and versatile polarization converters, has great potential for applications in polarization-controlled devices and is believed to be extendable to higher frequency regimes.

## Introduction

The polarization of electromagnetic (EM) waves is crucial in electromagnetic wave propagation, which has aroused increasing attention and interest, especially in the visible spectrum^1–3^. How to optionally regulate the polarization form of EM waves in the process of propagation is an essential and persistent research topic that has fascinating application prospects for the range from microwaves to visible light^4–8^, such as spectroscopic analysis^9,10^, sensing^11,12^, and polarization imaging^13^. Conventional approaches to manipulate the polarization state of light using birefringent crystals and grating structures are subject to bulky waveplates^14–16^; thus, the inherent defects of conventional polarizers include their large geometry and the low efficiency of natural materials, which make it difficult to meet the current miniaturization requirements of microwave and optical system integration. Metamaterials (MMs) are artificially engineered planar materials that typically have subwavelength periodic structures^17,18^ and exhibit EM properties that do not exist in nature, such as negative refraction^19,20^, perfect absorption^17,21^, and electromagnetic cloaking^22,23^. Metasurfaces (MSs) are two-dimensional MMs that not only possess exotic EM characteristics that do not exist in natural metamaterials but also have design, fabrication and integration advantages compared to MMs.

In recent years, MSs have become promising alternatives to conventional polarization manipulation devices due to their extreme wavefront and low-profile polarization control characteristics^2^. Myriad MS-based polarization conversion devices with various structures, including typical SSR units, have been proposed theoretically and experimentally for waves ranging from the microwave to optical frequencies, such as multiband or broadband linearly orthogonal polarization converters for reflection or transmission EM waves^24–27^, circular cross-polarization converters^28,29^, and linear-circular polarization converters^30–32^. Hao et al. reported an I-shaped structure achieving 90° polarization rotation in reflection mode with a high efficiency conversion ratio at two different frequencies for linearly polarized waves working in the microwave frequency^33^. Subsequently, a broadband reflection cross-polarization conversion was realized using cut-wire MSs in microwave and terahertz frequency bands^34–36^. Additionally, novel metallic helical MMs were introduced as polarizers that could achieve asymmetric, broadband circular-polarization conversion^37^. Various structures are used to achieve highly efficient and multiband MM-based linear to circular polarization conversion, such as SRR^38,39^, Q-shaped^40^ and twisted Hilbert-shaped chiral MSs^41^. Although MS-based polarization conversion devices have dramatically greater bandwidth and efficiency than conventional polarization filters, one essential issue with MSs-based polarization conversion in previous contributions is the independence of reflected and transmitted waves. The polarization states can be manipulated with the MS converters of the reflected waves without considering the transmission process since the metallic ground plane can block the transmission of EM waves. In addition, transmitted polarization converters can convert transmitted EM waves while neglecting reflected EM waves. Therefore, simultaneous manipulation of reflected and transmitted waves under different polarized mode illumination is still a problem in practical applications.

In this paper, we numerically and experimentally present a two-layer MS based on typical split ring resonators (SRRs) to manipulate the polarization states of both reflected and transmitted waves. The results show that either x- or y-polarized waves normally incident are reflected and transmitted into their orthogonal components across the proposed MS. The proposed MS has a high PCR, above 90%, at both normal and oblique incidences. For an x-polarized incident wave, the MS converts the reflected wave into a y-polarized wave at *f*_1_ = 3.76 GHz and *f*_2_ = 9.74 GHz and can convert the transmitted wave into a y-polarized wave at *f*_1_ = 3.36 GHz, *f*_2_ = 7.66 GHz, and *f*_3_ = 11.25 GHz. When a y-polarized wave is incident, the reflected wave can be converted into an x-polarized wave at *f*_1_ = 4.64 GHz and *f*_2_ = 11.25 GHz, and the transmitted wave can be converted into an x-polarized wave at *f*_1_ = 4.19 GHz, *f*_2_ = 7.78 GHz, and *f*_3_ = 13.19 GHz. More importantly, the performance of the angular tolerance improves the device’s performance against the incidence and reduces the requirements of the optical components in the system. The designed MS has potential applications in EM polarization control and stealth systems.

## Design and characteristics

**Figure 1:**
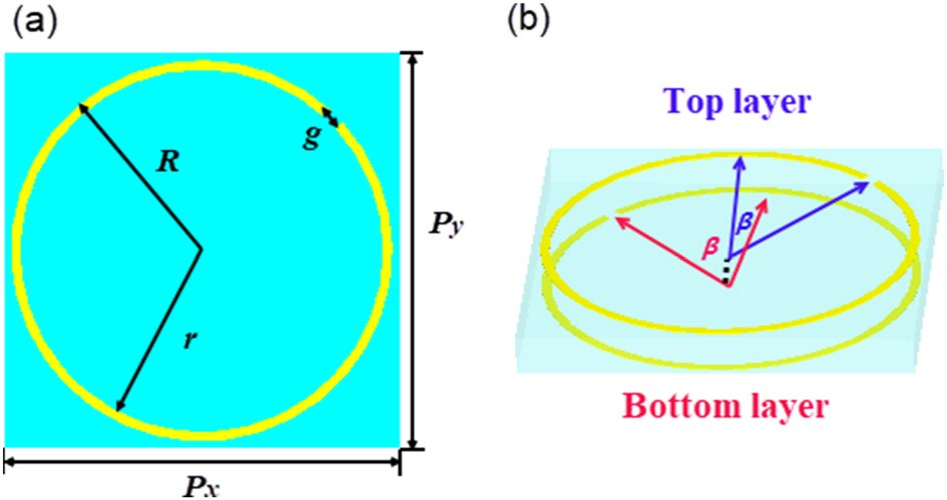
(**a**) The front view of structure in the *xoy* plane. For the top layer of copper SRR inlaid on the F4B substrate, the specific geometrical parameters of the structure are *R* = 4.8 mm, *r* = 4.56 mm, *g* = 0.4 mm, and *P*_*x*_ = *P*_*y*_ = 10 mm. (**b**) Schematic of the structure. The two identical copper layers are rotated by an angle of 2*β*, and *β* = 45° in the simulations.

Figure 1 shows the designed structure, which consists of two identical typical split rings separated by a substrate lamina. The top layer is shown in Fig. 1a, and the gap of the split ring is cut along the middle line between the x-axis and the y-axis. As shown in Fig. 1b, the angle (2*β*) of two gaps between the top layer and bottom layer is 90°. The substrate lamina is F4B with a thickness of 1 mm, and the complex permittivity is 2.65 with a dielectric loss tangent of 0.001. The split ring is made of copper with a thickness of 0.035 mm and a conductivity of 5.8 × 10^7^ S/m.

**Figure 2:**
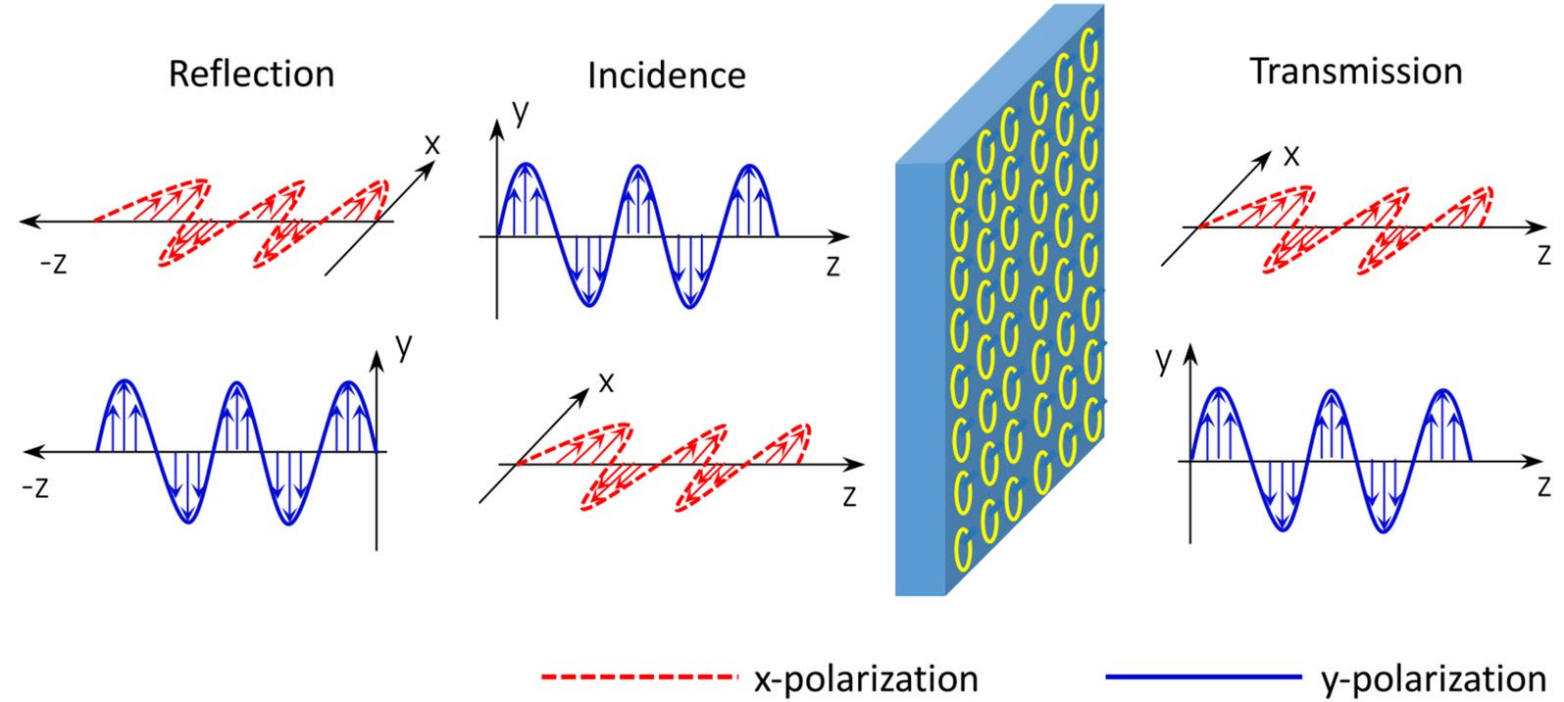
Operating principle diagram of the polarization conversion of the proposed MS. An x-polarized incident wave can be reflected and transmitted in the form of a y-polarized wave at the working frequencies, and a y-polarized wave can be reflected and transmitted as an x-polarized wave at the working frequencies. That is, the reflected wave and transmitted waves are converted into the orthogonal waves at the working frequencies. The red dotted line indicates the x-polarized wave, and the blue real line indicates the y-polarized wave.

Figure 2 shows the operating principle of the polarization conversion for the proposed MS. Such a simple MS can provide dual-polarization manipulation of reflected and transmitted waves simultaneously. As shown in Fig. 2, when x-polarized waves are incident to the designed MS, they can be converted into y-polarized waves in reflection mode and y-polarized waves in transmission mode. A y-polarized wave incident to the designed MS can be converted into its orthogonal polarized wave in reflection mode and simultaneously into an x-polarized wave in the transmission case. The above working processes demonstrates that the designed MS can simultaneously regulate reflected waves and transmitted waves with different functionalities.

## Results and discussion

First, we define the reflection matrix ***R*** and transmission matrix ***T*** as^42^:

1 $$ {\varvec{R}} = \left( {\begin{array}{*{20}c} {{\varvec{r}}_{xx} } & {{\varvec{r}}_{xy} } \\ {{\varvec{r}}_{yx} } & {{\varvec{r}}_{yy} } \\ \end{array} } \right),\;{\varvec{T}} = \left( {\begin{array}{*{20}c} {{\varvec{t}}_{xx} } & {{\varvec{t}}_{xy} } \\ {{\varvec{t}}_{yx} } & {{\varvec{t}}_{yy} } \\ \end{array} } \right) $$

Herein, x and y represent x- and y-polarized waves, respectively. *r*_*xx*_ and *r*_*yx*_ represent the reflectances of copolarization and cross-polarization when an x-polarized wave is incident to the MS, respectively. *r*_*yy*_ and *r*_*xy*_ represent the reflectances of copolarization and cross-polarization when y-polarized waves are incident to the MS, respectively. Correspondingly, the transmitted waves are defined in the same way. The PCR is used to reveal the polarization conversion performance of the proposed MS, which can be defined as^33,43^:

2 $$ {\text{Reflection}}{:}\;{\text{PCR}}_{x} = \frac{{r_{yx}^{2}} }{{r_{yx}^{{2}} + r_{xx}^{{2}} }},\;{\text{PCR}}_{y} = \frac{{r_{xy}^{{2}} }}{{r_{xy}^{{2}} + r_{yy}^{{2}} }} $$

3 $$ {\text{Transmission:}}\;{\text{PCR}}_{x} = \frac{{t_{yx}^{{2}} }}{{t_{yx}^{{2}} + t_{xx}^{{2}} }},\;{\text{PCR}}_{y} = \frac{{t_{xy}^{{2}} }}{{t_{xy}^{{2}} + t_{yy}^{{2}} }} $$

Herein, the transmittance is not considered when calculating the PCR of reflection polarization, and similarly, the reflectance is neglected when calculating the PCR of transmission polarization. The advantage of this approach is that we can independently obtain the energy conversions of reflected and transmitted waves. The polarization azimuth rotation angle *Ψ* can be calculated by the following formula^43,44^:

4 $$ \Psi = \frac{{1}}{{2}}{\text{atan}}\frac{{{2}p{\text{cos}}\varphi }}{{{1} - p^{2}} } $$

where *p* = *r*_*ij*_/*r*_*jj*_ or *p* = *t*_*ij*_/*t*_*jj*_, *φ* represents the phase difference between the cross-polarization coefficient and copolarization coefficient in reflection or transmission cases.

**Figure 3:**
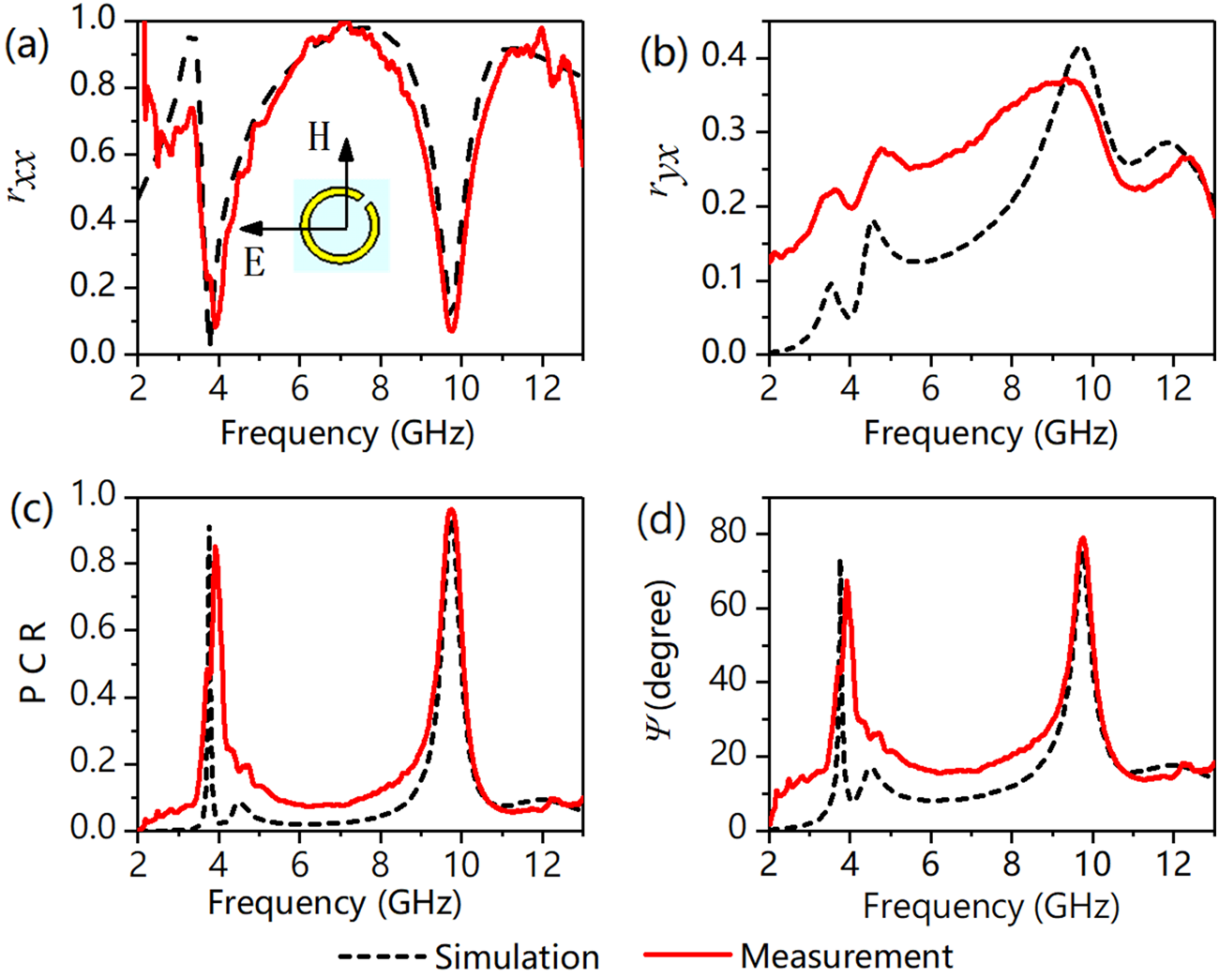
(**a**) Copolarization reflectance of *r*_*xx*_, (**b**) cross-polarization reflectance of *r*_*yx*_, (**c**) polarization conversion ratio (PCR), and (**d**) polarization rotation angle of *Ψ*. The inset of (**a**) depicts the orientation of the fields with respect to the structure, i.e., the directions of the E-field and H-field along the x- and y-axes, respectively.

Figure 3 depicts the simulated and experimental reflectances under an x-polarized incident wave. Figure 3a, b show that *r*_*xx*_ is 0.02 and 0.01 and *r*_*yx*_ is 0.09 and 0.41 at *f*_1_ = 3.76 GHz and *f*_2_ = 9.74 GHz, respectively. We can see from Fig. 3b, c that the *r*_*yx*_ of the converted y-polarized wave is slightly low, but the PCR is larger than 0.9 at *f*_1_ = 3.76 GHz, which means that more than 90% of the reflected energy has already been converted into y-polarized waves. The polarization azimuth rotation angle Ψ calculated from the simulation and experimental results are shown in Fig. 3d.The Ψ under the x-polarized incident wave is 73.4° and 75.6° at *f*_1_ = 3.76 GHz and *f*_2_ = 9.74 GHz, respectively. This means that the polarization angle of the reflected wave is rotated by 73.4° and 75.6° relative to the incident wave. The experimental results verified the correctness of the simulation results. Specifically, the cross-polarization reflection coefficient in the experiment is significantly different from that in the simulation, and the experimental result is larger than that in the simulation at 2–9 GHz. This error is generated during the experiment and is inevitable, which is explained in detail in the experimental section.

**Figure 4:**
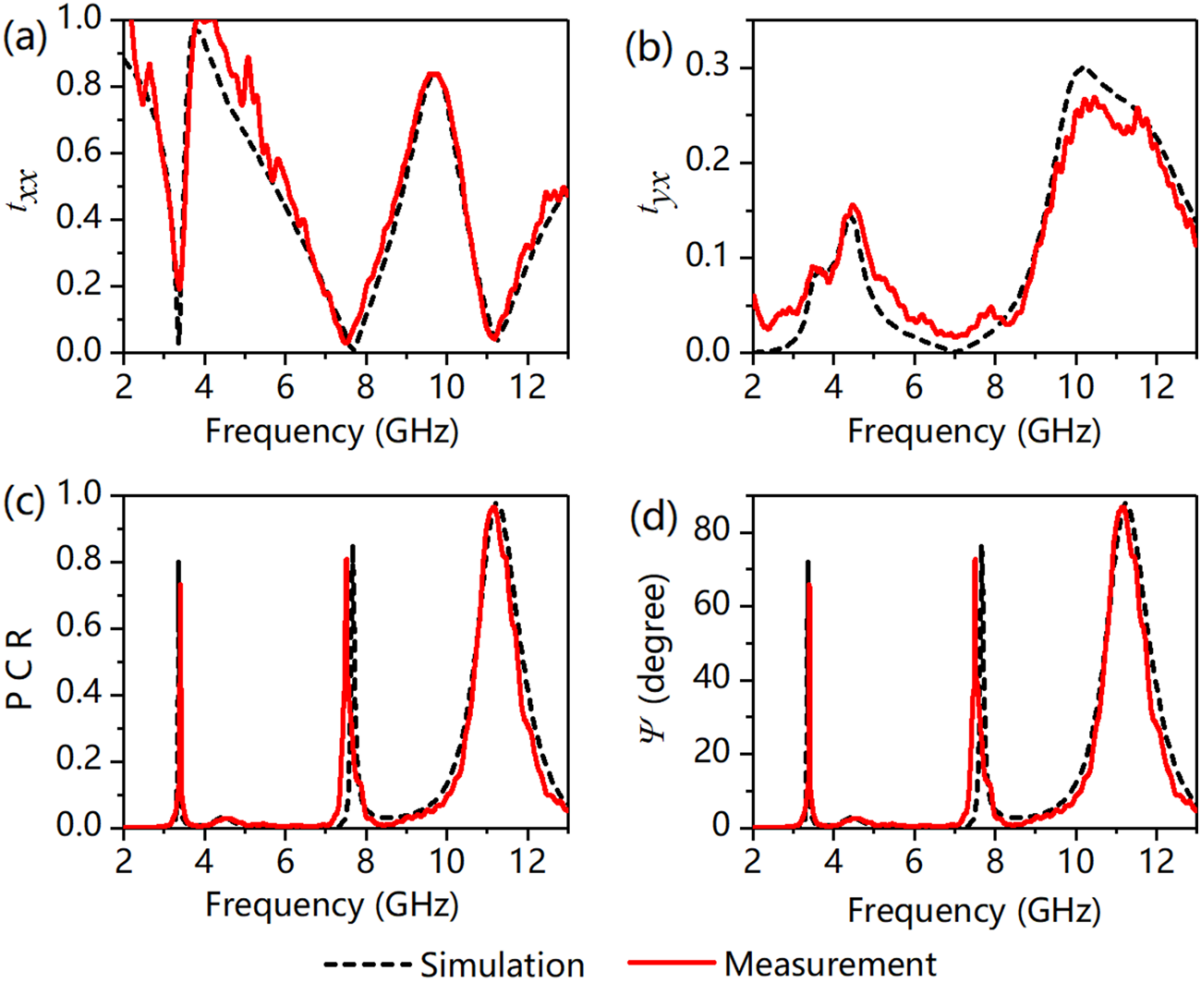
(**a**) Copolarization transmittance of *t*_*xx*_, (**b**) cross-polarization transmittance of *t*_*yx*_, (**c**) polarization conversion ratio (PCR), and (**d**) polarization rotation angle of *Ψ*.

Figure 4 shows the simulated and experimental transmission results when an x-polarized wave is incident. We can see from Fig. 4a, b that *t*_*xx*_ approaches zero and *t*_*yx*_ is approximately 0.05, 0.04, and 0.28 at *f*_1_ = 3.36 GHz, *f*_2_ = 7.66 GHz, and *f*_3_ = 11.25 GHz, respectively. The PCR is larger than 0.8 and Ψ is larger than 75° at these three frequencies, which is shown in Fig. 4c, d. Therefore, it is proven that the transmitted wave is nearly converted into its orthogonal component through the designed MS.

**Figure 5:**
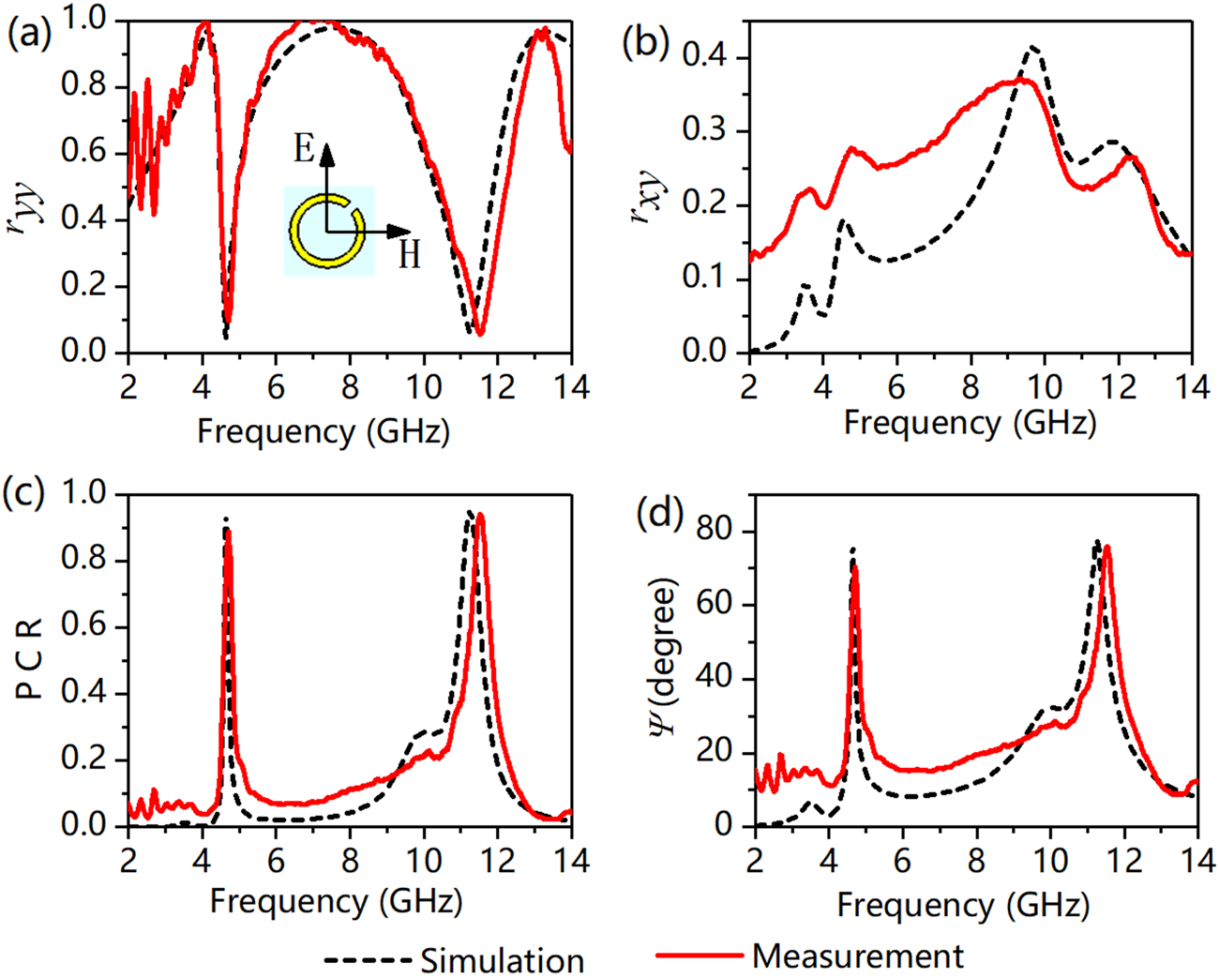
Results of reflected waves under a y-polarized incident wave. The inset of (**a**) depicts the orientation of the fields with respect to the structure, i.e., the directions of the H-field and E-field along the x- and y-axes, respectively. (**a**) Copolarization reflectance of *r*_*yy*_, (**b**) cross-polarization reflectance of *r*_*xy*_, (**c**) polarization conversion ratio (PCR), and (**d**) polarization rotation angle of *Ψ*.

**Figure 6:**
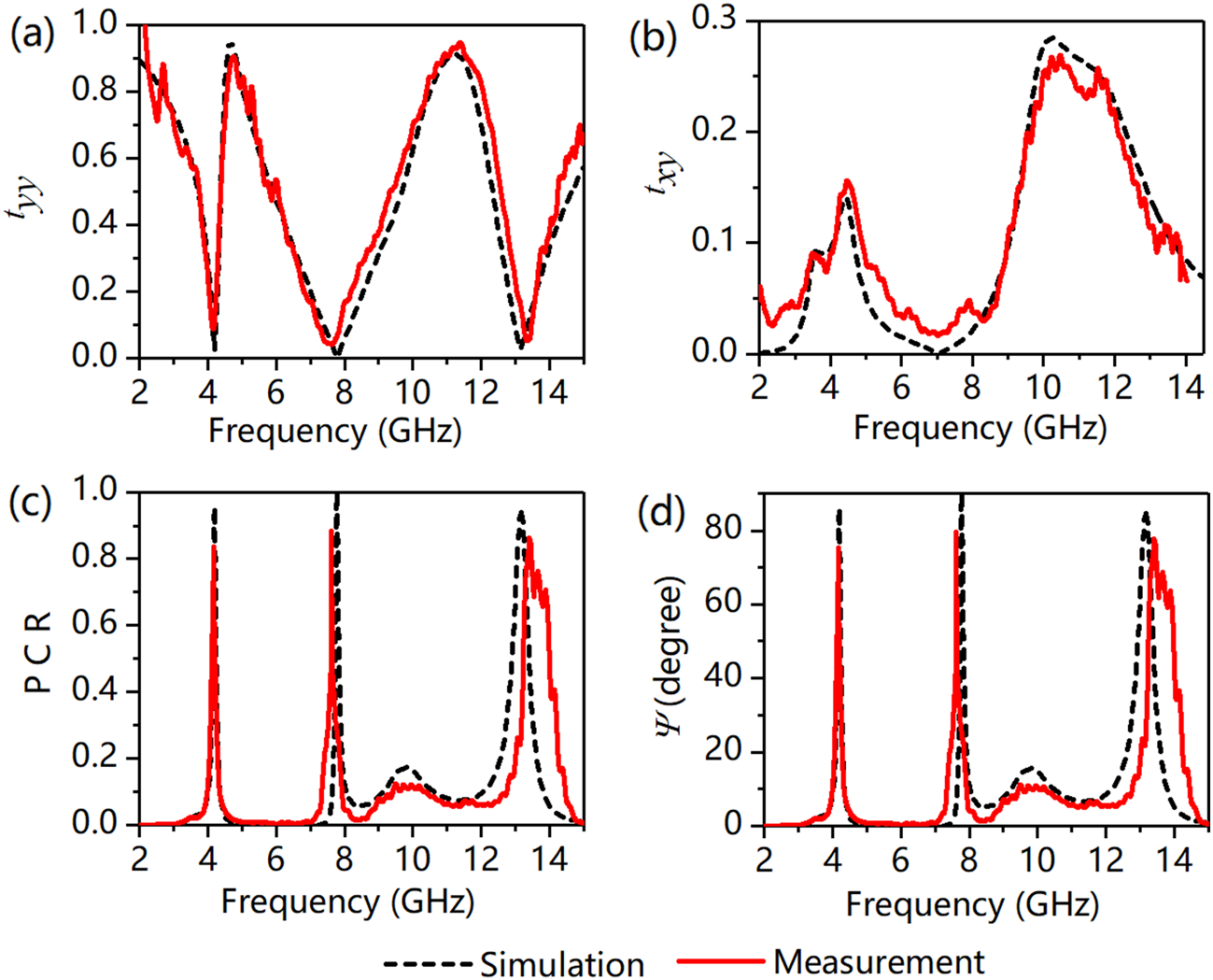
(**a**) Copolarization transmittance of *t*_*yy*_, (**b**) cross-polarization transmittance of *t*_*xy*_, (**c**) polarization conversion ratio (PCR), and (**d**) polarization rotation angle of *Ψ*.

In what follows, we consider the polarization properties of the designed MS under a y-polarized incident wave. From Fig. 5a, b, we can see that *r*_*yy*_ is 0.04 and 0.06 at *f*_1_ = 4.64 GHz and *f*_2_ = 11.25 GHz and *r*_*xy*_ is 0.19 and 0.27 at these two frequencies, respectively. Accordingly, the PCR and *Ψ* are calculated and are shown in Fig. 5c, d. We can see that the PCR is greater than 0.95 at *f*_1_ = 4.64 GHz and *f*_2_ = 11.25 GHz, and *Ψ* of the *y*-polarized incident wave is 75.9° and 77.4° at these two frequencies, respectively. The simulated and experimental transmission spectra under a normally incident y-polarized wave are shown in Fig. 6. We can see that *t*_*yy*_ is almost 0, while *t*_*xy*_ exists at *f*_1_ = 4.20 GHz, *f*_2_ = 7.78 GHz, and *f*_3_ = 13.17 GHz. These above results demonstrate that polarization conversion is realized at these three frequencies. As shown in Fig. 6c, d, the PCR and *Ψ* under a y-polarized incident wave confirmed that the MS can convert the incident wave into its cross-component wave at 4.20 GHz, 7.78 GHz, and 13.17 GHz.

**Figure 7:**
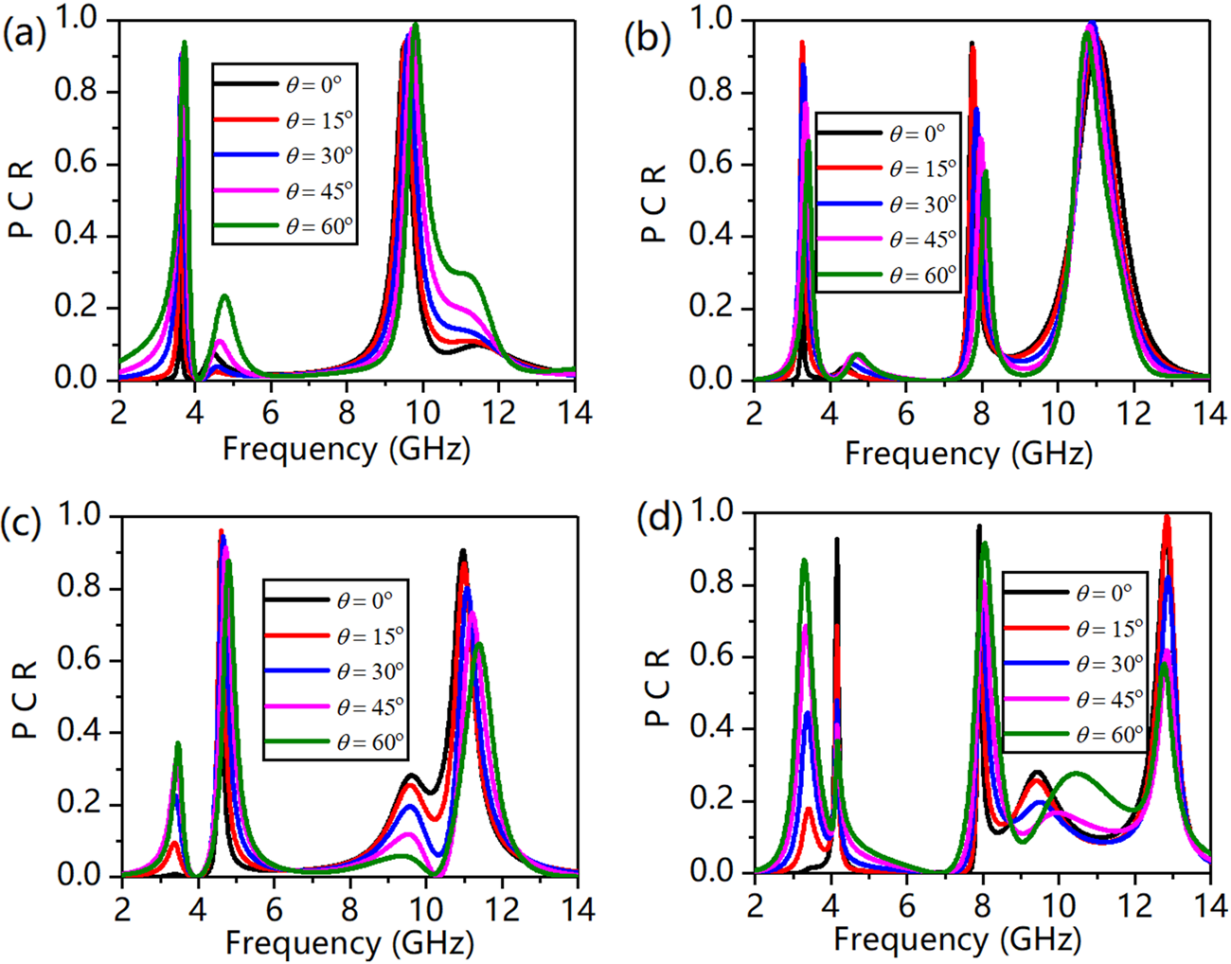
(**a**) PCR of reflection under x-polarization, (**b**) PCR of transmission under x-polarization, (**c**) PCR of reflection under y-polarization, and (**d**) PCR of transmission wave under y-polarization.

**Figure 8:**
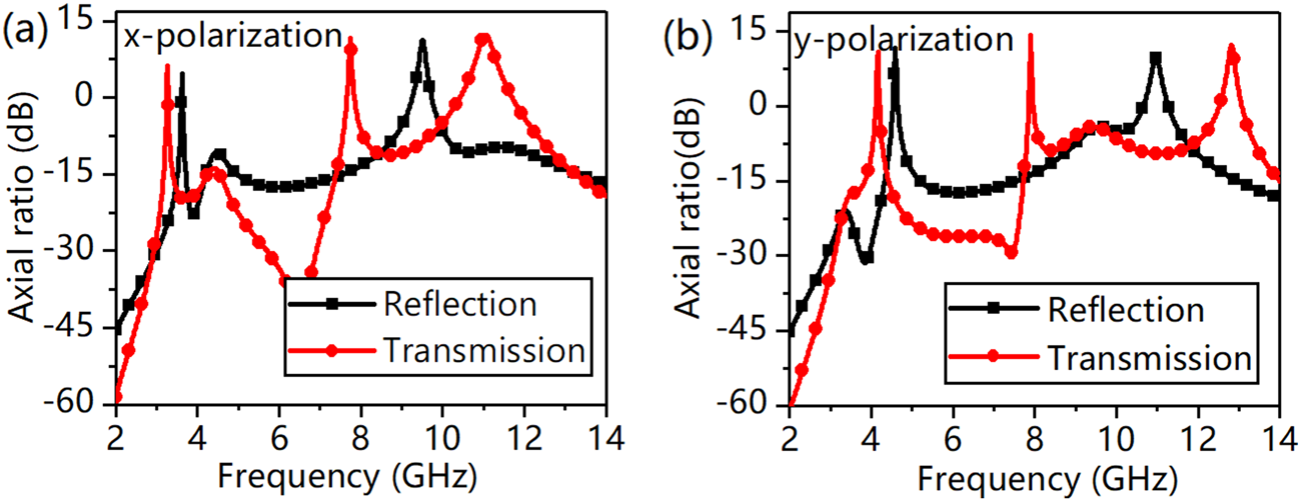
Axial ratio of the cross-polarization to copolarization, (**a**) the axial ratio of the reflection and transmission of an x-polarized incident wave, and (**b**) the axial ratio of the reflection and transmission of a y-polarized incident wave.

The designed MS is superior in that it can work at a wide oblique incident angle. Figure 7 shows the PCR at different incident angles ranging from 0° to 60° with a step of 15°. We can see from Fig. 7a, b that under an x-polarized incident wave, when the incident angle increases to 60°, the PCR is almost unaffected in both reflection mode and transmission mode. However, the PCR in both the reflection and transmission modes decreases monotonically when the incident angle increases from 0° to 60° under a y-polarized incident wave, as shown in Fig. 7c, d. Specifically, the PCR decreases with increasing incident angle from 0° to 60° under an x-polarized incident wave at 3.36 GHz and 7.67 GHz because the transmittance at these two frequencies is so small that a slight deviation can cause a change. This phenomenon does not appear with a y-polarized incident wave because the response of magnetic coupling between two layers is desirable for y-polarization. For an x-polarized incident wave, the direction of the magnetic field along the y-axis can effectively stimulate circular currents at arbitrary oblique incident angles. In contrast, in the case of a y-polarized incident wave, the magnetic field is along the x-axis, and the magnetic field is incapable of effectively exciting the circular currents at arbitrary oblique incident angles^44^. To allow a better view of the polarization conversion effect, the axial ratio is shown in Fig. 8. The value of the axial ratio is 4.59 dB at 3.76 GHz, 11.31 dB at 9.74 GHz, 6.18 dB at 3.36 GHz, 11.72 dB at 7.66 GHz, 12.30 dB at 11.25 GHz for an x-polarized incident wave; 11.79 dB at 4.64 GHz, 9.84 dB at 11.25 GHz, 10.99 dB at 4.19 GHz, 14.20 dB at 7.78 GHz, 12.38 dB at 13.17 GHz for a y-polarized incident wave. We can see that the axial ratio is nearly higher than 5 dB at resonant frequencies where the PCR is almost larger than 90%.

**Figure 9:**
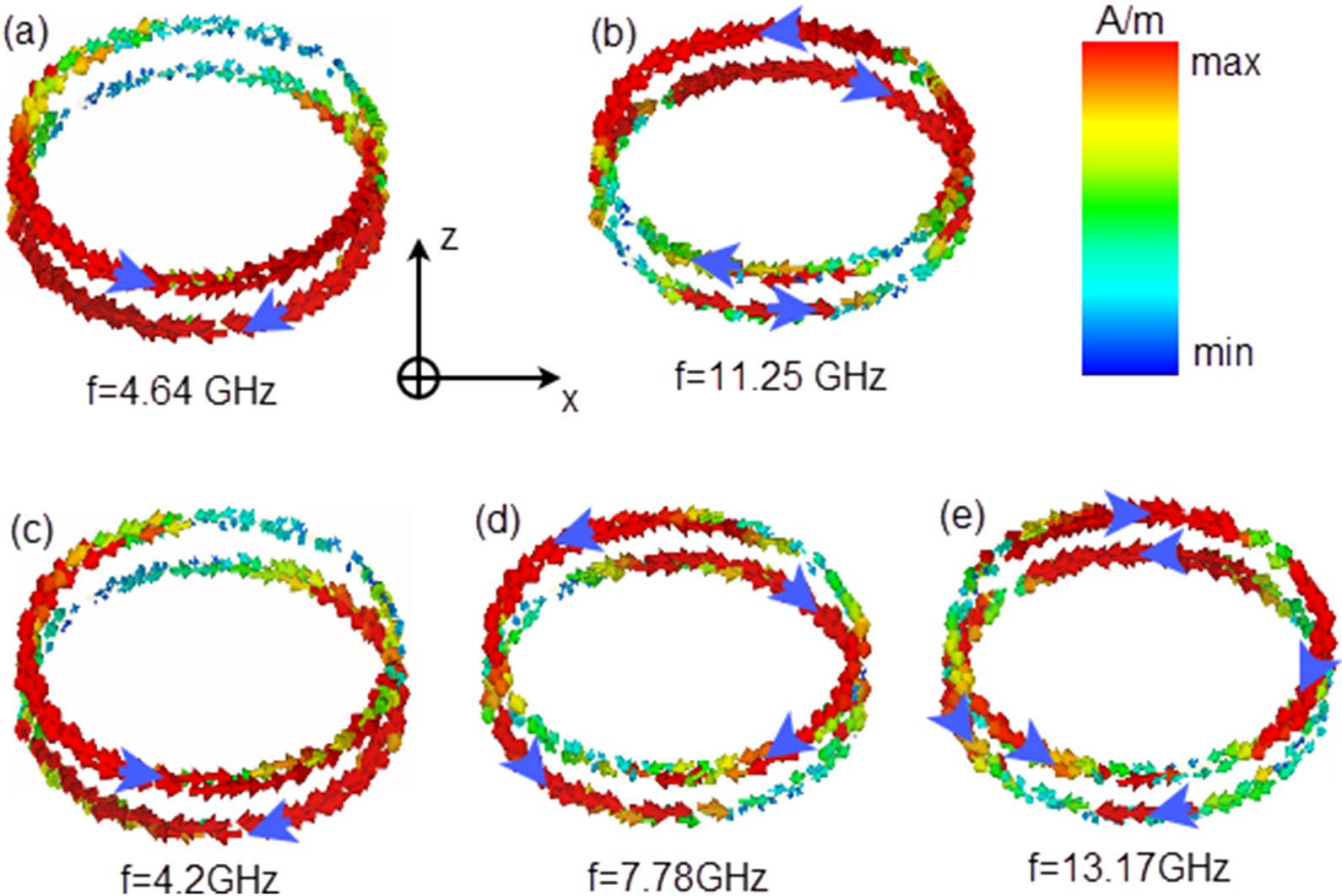
Surface current distributions. (**a**) and (**b**) are the reflected polarization resonances, and (**c**), (**d**) and (**e**) are the transmitted polarization resonances.

Herein, the physics of polarization conversion are investigated by using surface current distributions. We depict the surface current distributions in Fig. 9 and take a y-polarized wave as an example. Figure 9a shows the surface current distribution at 4.64 GHz; we can see from the figure that the magnetic dipole is excited along the y-axis; thus, the electric field corresponds to the x-axis, which means that the MS converts the y-polarized wave into an x-polarized wave at 4.64 GHz under the reflection situation. Compared with the frequency of 1.64 GHz, two magnetic dipoles are excited along the y-direction at 11.25 GHz, as shown in Fig. 9b, and it is also indicated that the incident y-polarized wave is converted into an x-polarized wave. The surface current distributions at three resonant frequencies along the z-axis for the transmitted wave are shown in Fig. 9c–e. The excited magnetic fields come from the surface current mainly along the y-axis, which indicates that the incident y-polarized wave is converted into an x-polarized wave after transmission at the three resonant points. The difference is that as the frequency increases, the number of excited magnetic dipoles gradually increases. It is well understood from the transmission line theory that as the resonant frequency increases, the capacitance and inductance that generate resonance decrease, and the corresponding current loop that forms the magnetic dipole gradually decreases.

## Conclusion

In conclusion, we demonstrated the design and experimental characteristics of a bilayered metasurface with polarization conversion of reflection waves and transmission waves. The designed metasurface can easily convert both the reflected wave and transmitted wave of x- and y-polarized waves into their orthogonal counterparts. The simulation results show that the PCR is greater than 90% in both x- and y-polarization. The measured results from microwave experiments verified the simulated results. The proposed device has potential applications in many fields related to polarization manipulation. Multiple layers and different structures could be investigated in future research, which has the potential to provide improved bandwidth and efficiency.

### Simulations

The numerical simulations were calculated by CST MICROWAVE STUDIO. The unit cell boundary conditions are applied in the x- and y-directions to mimic infinite boundaries; the open (add space) boundary condition is set in the z-direction to represent the propagation of EM waves. The port number of Floquet modes is set to 2 for both TE (x-polarization) and TM (y-polarization) waves; thus, the reflection and transmission parameters of co- and cross-polarization can be calculated simultaneously.

### Fabrication and experiments

**Figure 10:**
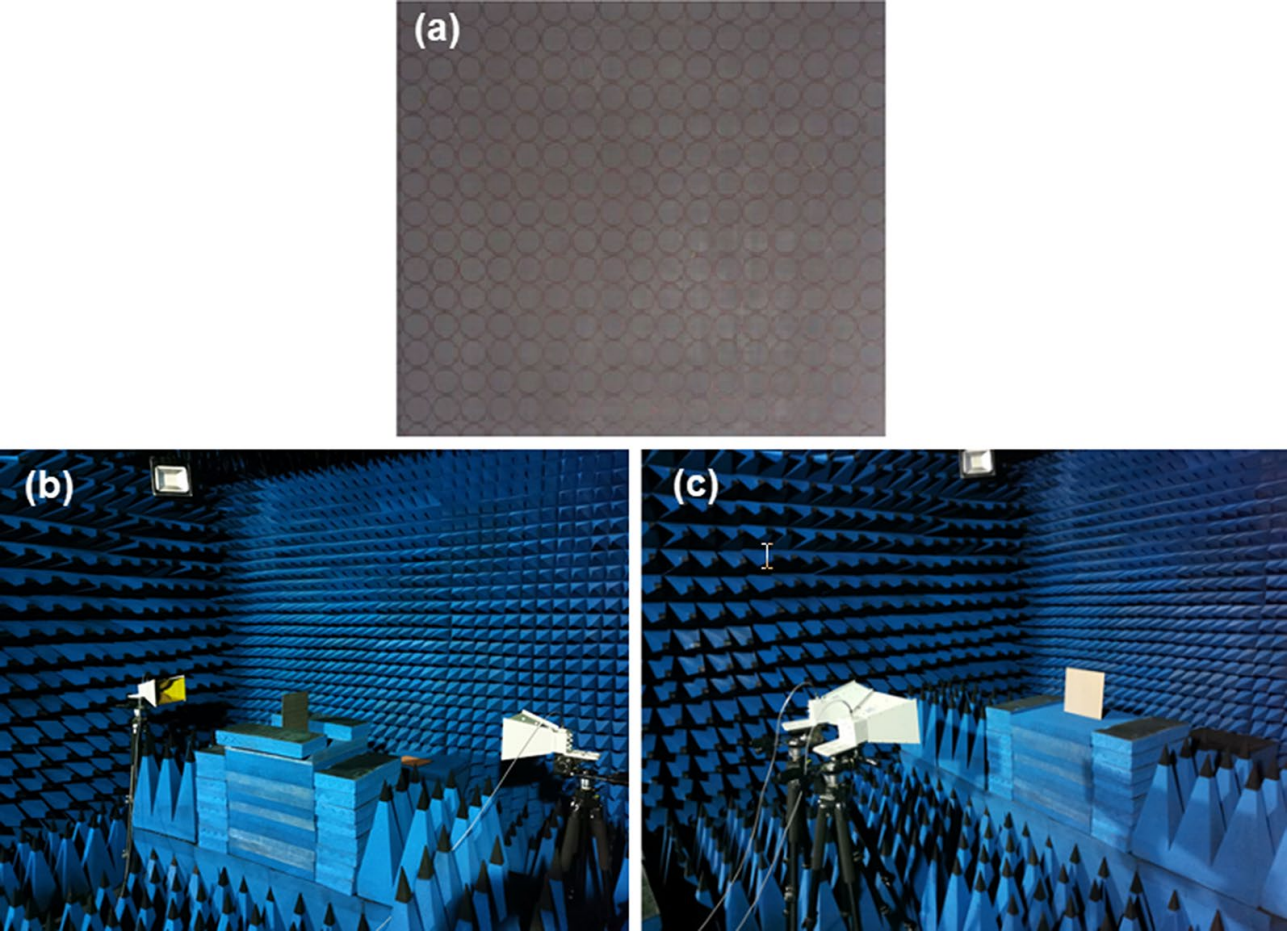
(**a**) Photograph of the fabricated sample; (**b**, **c**) photographs of the experimental environment.

For the measurements, the metasurface was fabricated with geometry identical to that of the simulations employing standard printed circuit board (PCB) technology. The experimental sample is shown in Fig. 10a. The reflection and transmission parameters were measured by a couple of identical standard broadband horn antennas (1–18 GHz) connected to the vector network analyser (Agilent E8362B) via cables in the anechoic chamber, as shown in Fig. 10b, c. To measure the reflection coefficients, the sample was placed on the same side of two horns, and a copper plate the same size as the sample was used for normalization. Horn 1 was first fixed in the horizontal direction to emit a y-polarized wave, which was reflected, and horn 2 was placed in the horizontal and vertical directions to receive the reflected wave with co- and cross-polarization reflection coefficients; then, horn 1 was rotated in the vertical direction to emit an x-polarized wave, and horn 2 received co- and cross-polarized waves in the same way. The distance between antennas and sample was 2 m to avoid the near field effect. A metal plate the same size as the sample was used for calibration before the test. To measure the transmission coefficients, the convertor was placed in the middle of the two horns, and the co- and cross-polarization transmittances with different polarizations could be tested by rotating the direction of the two antennas. Herein, we have to mention that the disparity of the cross-polarization coefficient between simulation and experiment was caused the inherent setting of the experimental platform, especially in terms of the cross-polarization reflection coefficients. The cross-polarization coefficient was tested by rotating the receiving antenna by 90° after calibration by a copolarization antenna; thus, the value of the calibration was slightly different when we rotated the antenna due to the inevitable change in the incident angle and the centre position of the receiving antenna.
